# Supplementary material for: Streptococcus gordonii Supragingival Bacterium Oral Infection-Induced Periodontitis and Robust miRNA Expression Kinetics
Source: Int J Mol Sci. 2024 Jun 5;25(11):6217. doi: 10.3390/ijms25116217 (PMC11172800; doi:10.3390/ijms25116217)
Supplement: Supplementary file 1 [file ijms-25-06217-s001.zip › ijms-3015440-supplementary.pdf]

**_Streptococcus gordonii_ supragingival bacterium oral infection-induced periodontitis and robust miRNA expression kinetics**

Chairmandurai Aravindraja [1,†,†], Syam Jeepipalli [1,†], William Duncan [2], Krishna Mukesh Vekariya [1], Shaik O. Rahaman [3], Edward K. L. Chan [4] and Lakshmyya Kesavalu [1,4]*

[1] Department of Periodontology, College of Dentistry, University of Florida, Gainesville, FL 32610, USA; aravindrchairman@ufl.edu (C.A.); sjeepipalli@dental.ufl.edu (S.J.); kvekariya@ufl.edu (K.M.V.);
[2] Department of Community Dentistry, College of Dentistry, University of Florida, Gainesville, FL 32610, USA; duncanw@ufl.edu
[3] Department of Nutrition and Food Science, University of Maryland, College Park, MD 20742, USA
[4] Department of Oral Biology, College of Dentistry, University of Florida, Gainesville, FL 32610, USA; echan@dental.ufl.edu
[†] These authors contributed equally to this work.
[†] Present address: Department of Neurology, College of Medicine, University of Florida, Gainesville, FL 32610, USA.
*Correspondence: kesavalu@dental.ufl.edu (L.K.); Tel.: +1-352-273-6500 (L.K.).

**Supplementary information**

**Table S1.** *S. gordonii* DL-1 PCR test for Bacterial dissemination to distal organs.

| Group/Bacteria/Infection (Weeks) | PCR Positive samples (n=10) | | | | | |
|---|---|---|---|---|---|---|
| | Heart | Lungs | Brain | Liver | Kidney | Spleen |
| Group I/ *S. gordonii* DL1 [8 weeks] | 0/10 | 0/10 | 0/10 | 0/10 | 0/10 | 0/10 |
| Group I/ *S. gordonii* DL1 [16 weeks] | 0/10 | 0/10 | 0/10 | 0/10 | 0/10 | 0/10 |
| Group III/ Sham-infection [8 weeks] | 0/10 | 0/10 | 0/10 | 0/10 | 0/10 | 0/10 |
| Group III/ Sham-infection [16 weeks] | 0/10 | 0/10 | 0/10 | 0/10 | 0/10 | 0/10 |

**Table S2.** List of miRNAs DE upregulated during 8 weeks of *S. gordonii* infection.

| miRNA | Fold Change | p value |
|---|---|---|
| miR-m59-2 | 1.47 | 0.0002 |
| mghv-miR-M1-3 | 1.47 | 0.0013 |
| mghv-miR-M1-2 | 1.43 | 0.0012 |
| mcmv-miR-m107-1-5p | 1.41 | 0.0007 |
| mghv-miR-M1-5 | 1.41 | 0.0008 |
| mcmv-miR-m88-1 | 1.39 | 0.0003 |
| mcmv-miR-m108-1 | 1.39 | 0.0016 |
| mcmv-miR-M55-1 | 1.37 | 0.0007 |
| miR-683 | 1.37 | 0.0210 |
| miR-1964 | 1.36 | 0.0014 |
| miR-468 | 1.36 | 0.0019 |

| | | |
|---|---|---|
| miR-380-5p | 1.36 | 0.0021 |
| miR-590-5p | 1.36 | 0.0022 |
| miR-448 | 1.36 | 0.0022 |
| miR-1928 | 1.36 | 0.0023 |
| miR-883b-5p | 1.36 | 0.0078 |
| miR-148a | 1.36 | 0.0495 |
| miR-320 | 1.35 | 0.0012 |
| miR-1893 | 1.35 | 0.0042 |
| miR-1892 | 1.35 | 0.0042 |
| miR-1895 | 1.35 | 0.0051 |
| miR-338-5p | 1.35 | 0.0073 |
| miR-764-5p | 1.35 | 0.0111 |
| mcmv-miR-M23-1-3p | 1.35 | 0.0125 |
| miR-200c | 1.35 | 0.0431 |
| miR-652 | 1.34 | 0.0043 |
| miR-467e | 1.34 | 0.0052 |
| miR-883a-3p | 1.34 | 0.0106 |
| miR-1943 | 1.34 | 0.0106 |
| miR-3474 | 1.34 | 0.0135 |
| miR-467f | 1.33 | 0.0025 |
| mmu-miR-105 | 1.33 | 0.0041 |
| mghv-miR-M1-8 | 1.33 | 0.0041 |
| miR-883a-5p | 1.33 | 0.0063 |
| miR-1194 | 1.33 | 0.0068 |
| miR-464 | 1.33 | 0.0087 |
| miR-669a | 1.33 | 0.0087 |
| miR-760 | 1.33 | 0.0091 |
| mghv-miR-M1-7-5p | 1.33 | 0.0104 |
| miR-761 | 1.33 | 0.0139 |
| miR-125b-3p | 1.32 | 0.0028 |
| miR-129-5p | 1.32 | 0.0046 |
| miR-33 | 1.32 | 0.0046 |
| miR-467a | 1.32 | 0.0056 |
| miR-1197 | 1.32 | 0.0064 |
| miR-463 | 1.32 | 0.0081 |
| mcmv-miR-m107-1-3p | 1.32 | 0.0082 |
| miR-337-5p | 1.32 | 0.0147 |
| miR-882 | 1.32 | 0.0227 |
| miR-1946a | 1.32 | 0.0270 |
| miR-695 | 1.31 | 0.0019 |
| miR-362-3p | 1.31 | 0.0029 |
| miR-2146 | 1.31 | 0.0046 |
| miR-490 | 1.31 | 0.0054 |

| | | |
|---|---|---|
| mghv-miR-M1-1 | 1.31 | 0.0056 |
| miR-1962 | 1.31 | 0.0058 |
| mcmv-miR-M23-1-5p | 1.31 | 0.0071 |
| miR-493 | 1.31 | 0.0088 |
| miR-134 | 1.31 | 0.0094 |
| miR-488 | 1.31 | 0.0103 |
| miR-879 | 1.31 | 0.0116 |
| miR-668 | 1.31 | 0.0172 |
| miR-1945 | 1.31 | 0.0223 |
| mcmv-miR-m21-1 | 1.31 | 0.0269 |
| miR-3473 | 1.3 | 0.0012 |
| miR-146b | 1.3 | 0.0012 |
| miR-1956 | 1.3 | 0.0031 |
| miR-431 | 1.3 | 0.0045 |
| miR-421 | 1.3 | 0.0073 |
| miR-684 | 1.3 | 0.0120 |
| mcmv-miR-m108-2-5p.1 | 1.3 | 0.0152 |
| miR-142-3p | 1.3 | 0.0194 |
| mcmv-miR-m01-3 | 1.3 | 0.0228 |
| miR-207 | 1.3 | 0.0312 |
| miR-1195 | 1.3 | 0.0327 |
| miR-574-5p | 1.29 | 0.0007 |
| miR-669i | 1.29 | 0.0067 |
| miR-708 | 1.29 | 0.0073 |
| miR-770-5p | 1.29 | 0.0096 |
| miR-184 | 1.29 | 0.0134 |
| miR-467g | 1.29 | 0.0182 |
| mcmv-miR-m01-1 | 1.29 | 0.0200 |
| miR-3472 | 1.29 | 0.0249 |
| miR-871 | 1.29 | 0.0258 |
| miR-1188 | 1.29 | 0.0352 |
| miR-346 | 1.29 | 0.0449 |
| miR-449a | 1.29 | 0.0467 |
| miR-875-3p | 1.28 | 0.0013 |
| miR-551b | 1.28 | 0.0033 |
| miR-542-5p | 1.28 | 0.0046 |
| miR-717 | 1.28 | 0.0054 |
| miR-32 | 1.28 | 0.0058 |
| miR-370 | 1.28 | 0.0083 |
| miR-202-5p | 1.28 | 0.0084 |
| miR-31 | 1.28 | 0.0113 |
| miR-433 | 1.28 | 0.0122 |
| miR-688 | 1.28 | 0.0127 |

| | | |
|---|---|---|
| miR-1958 | 1.28 | 0.0129 |
| miR-181b+mmu-miR-181d | 1.28 | 0.0132 |
| miR-1190 | 1.28 | 0.0148 |
| miR-7a | 1.28 | 0.0240 |
| miR-210 | 1.28 | 0.0258 |
| miR-491 | 1.28 | 0.0413 |
| miR-434-5p | 1.27 | 0.0060 |
| miR-384-3p | 1.27 | 0.0090 |
| miR-367 | 1.27 | 0.0101 |
| miR-694 | 1.27 | 0.0180 |
| miR-1966 | 1.27 | 0.0236 |
| miR-665 | 1.27 | 0.0413 |
| miR-2145 | 1.26 | 0.0045 |
| miR-541 | 1.26 | 0.0193 |
| miR-654-3p | 1.26 | 0.0254 |
| miR-675-5p | 1.26 | 0.0305 |
| miR-1941-5p | 1.26 | 0.0352 |
| miR-412 | 1.26 | 0.0352 |
| miR-682 | 1.26 | 0.0389 |
| miR-3471 | 1.26 | 0.0444 |
| miR-7b | 1.25 | 0.0070 |
| miR-501-5p | 1.25 | 0.0086 |
| miR-3470a+mmu-miR-3470b | 1.25 | 0.0145 |
| miR-484 | 1.25 | 0.0197 |
| miR-1963 | 1.25 | 0.0200 |
| mghv-miR-M1-4 | 1.25 | 0.0272 |
| miR-466k | 1.25 | 0.0273 |
| miR-1898 | 1.25 | 0.0275 |
| miR-669g | 1.25 | 0.0276 |
| miR-1967 | 1.24 | 0.0137 |
| miR-770-3p | 1.24 | 0.0156 |
| miR-873 | 1.24 | 0.0265 |
| miR-181c | 1.23 | 0.0032 |
| miR-423-3p | 1.23 | 0.0130 |
| miR-1965 | 1.23 | 0.0228 |
| miR-335-3p | 1.23 | 0.0477 |
| miR-3475 | 1.22 | 0.0325 |
| miR-369-5p | 1.22 | 0.0349 |
| miR-669j | 1.22 | 0.0463 |
| miR-425 | 1.21 | 0.0042 |
| miR-15b | 1.21 | 0.0082 |
| miR-300 | 1.21 | 0.0144 |

| | | |
|---|---|---|
| miR-130b | 1.21 | 0.0187 |
| miR-202-3p | 1.21 | 0.0434 |
| miR-1961 | 1.21 | 0.0435 |
| miR-2141 | 1.21 | 0.0460 |
| miR-1900 | 1.21 | 0.0484 |
| miR-674 | 1.2 | 0.0154 |
| miR-290-3p | 1.2 | 0.0202 |
| miR-214 | 1.2 | 0.0226 |
| miR-1959 | 1.2 | 0.0296 |
| miR-1196 | 1.19 | 0.0330 |
| miR-192 | 1.19 | 0.0423 |
| miR-331-3p | 1.18 | 0.0341 |
| miR-676 | 1.16 | 0.0265 |
| miR-574-3p | 1.16 | 0.0270 |
| miR-382 | 1.16 | 0.0363 |
| miR-191 | 1.15 | 0.0006 |
| miR-374 | 1.15 | 0.0199 |
| miR-23a | 1.15 | 0.0329 |
| miR-423-5p | 1.14 | 0.0073 |
| miR-106a+mmu-miR-17 | 1.14 | 0.0148 |
| miR-532-5p | 1.13 | 0.0001 |
| miR-125a-5p | 1.12 | 0.0480 |
| miR-93 | 1.11 | 0.0330 |

A total of 191 miRNAs DE upregulated during 8 weeks of *S. gordonii* infection.

**Table S3**. List of miRNAs DE downregulated during 8 weeks of *S. gordonii* infection.

| **miRNA** | **Fold Change** | **p value** |
|---|---|---|
| miR-133a | -1.82 | 0.0191 |
| miR-1224 | -1.75 | 0.0024 |
| miR-2135 | -1.73 | 0.0007 |
| miR-499 | -1.73 | 0.0115 |
| miR-378 | -1.56 | 0.0212 |
| miR-2133 | -1.55 | 0.0052 |
| miR-22 | -1.53 | 0.0009 |
| miR-361 | -1.36 | 0.0132 |
| miR-133b | -1.34 | 0.0457 |
| miR-30a | -1.33 | 0.0140 |
| miR-450a-5p | -1.32 | 0.0014 |
| miR-29c | -1.3 | 0.0098 |
| miR-30c | -1.26 | 0.0111 |
| miR-149 | -1.26 | 0.0314 |
| miR-30b | -1.25 | 0.0010 |

| | | |
|---|---|---|
| miR-101a | -1.23 | 0.0058 |
| miR-26a | -1.19 | 0.0068 |
| miR-26b | -1.14 | 0.0028 |
| miR-30d | -1.14 | 0.0323 |
| miR-145 | -1.13 | 0.0133 |
| miR-151-5p | -1.1 | 0.0427 |
| miR-350 | -1.09 | 0.0151 |

A total of 22 miRNAs DE downregulated during 8 weeks of *S. gordonii* infection.

**Table S4.** List of miRNAs DE downregulated during 16-weeks of *S. gordonii* infection.

| **miRs** | **Fold change** | **p-value** |
|---|---|---|
| miR-720 | -1.66 | $2.23 \times 10^{-6}$ |
| miR-1937c | -1.47 | $1.13 \times 10^{-4}$ |
| miR-2135 | -1.43 | $4.24 \times 10^{-4}$ |
| miR-326 | -1.41 | $3.15 \times 10^{-4}$ |
| miR-342-5p | -1.39 | $1.63 \times 10^{-4}$ |
| miR-1937a+mmu-miR-1937b | -1.39 | $7.76 \times 10^{-3}$ |
| miR-323-3p | -1.38 | $6.52 \times 10^{-4}$ |
| miR-362-3p | -1.37 | $1.28 \times 10^{-3}$ |
| miR-690 | -1.35 | $7.68 \times 10^{-3}$ |
| miR-125b-5p | -1.33 | $1.83 \times 10^{-3}$ |
| miR-140 | -1.32 | $4.01 \times 10^{-3}$ |
| miR-130a | -1.29 | $1.46 \times 10^{-3}$ |
| miR-1274a | -1.29 | $1.51 \times 10^{-3}$ |
| miR-322 | -1.29 | $1.35 \times 10^{-2}$ |
| miR-488 | -1.29 | $2.12 \times 10^{-2}$ |
| miR-10a | -1.28 | $1.36 \times 10^{-2}$ |
| miR-195 | -1.27 | $2.43 \times 10^{-3}$ |
| miR-19b | -1.27 | $2.48 \times 10^{-3}$ |
| miR-331-3p | -1.24 | $3.56 \times 10^{-3}$ |
| miR-669f | -1.24 | $1.97 \times 10^{-2}$ |
| miR-496 | -1.23 | $5.08 \times 10^{-3}$ |
| miR-2141 | -1.23 | $4.88 \times 10^{-2}$ |
| miR-99a | -1.21 | $1.71 \times 10^{-2}$ |
| miR-1968 | -1.21 | $3.10 \times 10^{-2}$ |
| miR-34a | -1.2 | $4.28 \times 10^{-2}$ |
| miR-151-3p | -1.19 | $1.63 \times 10^{-4}$ |
| miR-582-5p | -1.18 | $5.50 \times 10^{-3}$ |
| miR-107 | -1.18 | $2.37 \times 10^{-2}$ |
| miR-143 | -1.17 | $1.66 \times 10^{-3}$ |
| miR-15a | -1.12 | $9.99 \times 10^{-3}$ |
| miR-145 | -1.11 | $1.56 \times 10^{-2}$ |

| | | |
|---|---|---|
| miR-219 | -1.08 | $3.02\times10^{-2}$ |

A total of 32 miRNAs DE downregulated during 16 weeks of S. gordonii infection.

**Table S5.** Comparison of DE miRNAs between 8- and 16- weeks of *S. gordonii* infection and their reported functions, and target genes.

| miRNA | Fold Change | p value | Target Function | Number of Functional genes |
|---|---|---|---|---|
| miR-375 | 2.03 | 0.0464 | Down-regulated in oral squamous cell carcinoma [143] and salivary adenoid cystic carcinoma [144]. | 24 (e.g., *Med13, C1qbp, Mtpn, Wdr26, Sept2*) |
| miR-429 | 1.65 | 0.0397 | Reported as an inflammatory mediator in gingival cells and suggested as therapeutic target of oral inflammatory diseases [145]. Upregulated in neonatal peripheral blood and LPS-stimulated WI-38 cells [146]). | 48 (e.g., *Zeb1, Flt1, Map2, Plxnc1, Pkib*) |
| miR-205 | 1.41 | 0.0211 | Reported in the 8 weeks analysis. | |
| miR-210 | 1.36 | 0.0145 | Upregulated in periodontal disease and obesity-suffering subjects [99]. Elevated in the muscle samples of peripheral artery diseases and atherosclerosis obliterans [147]. Frequently elevated in multiple cancers such as HCC, prostate cancer, colorectal cancer and gastric cancer. | 47 (e.g., *Tcf7l2, Acvr1b, Ucp2, NFKB1, Bcl2*) |
| miR-200c | 1.32 | 0.0463 | Reduced levels observed in mice infected with LPS of *P. gingivalis* [148]. | 15 (e.g., *Atp5b, Pls3, Mycn, Zeb1, Thap4*) |
| **miR-126-5p** | 1.29 | 0.0228 | Upregulated in the *T. denticola*-induced periodontitis in the mice models [25]. Attenuates blood–brain barrier after ischemic stroke [149]. | 55 (e.g., *Rab3c, Il10rb, Lrrc32, H2-D1, Klhl31*) |
| miR-203 | 1.27 | 0.0347 | Reported in the 16 weeks analysis (Table 4) | |
| miR-767 | 1.25 | 0.0359 | Expressed in the heart tissue of the patients with myocardial infarction [150]. Downregulated in senescent skin fibroblasts and increased in senescent vascular endothelial cells [58]. | 13 (e.g., *Mcl1, Tmem178b, Chm, Ttc38, Lactb2*) |
| miR-590-5p | 1.24 | 0.0263 | Inhibiting pathological hypertrophy mediated heart failure [151]. Improved cardiac function in the mice suffered from myocardial infarction [152]. | 12 (e.g., *Kcnk6, Map3k1, Cyfip1, Rmnd5a, Tns1*) |
| miR-129-3p | 1.24 | 0.0315 | Reported in the 8 weeks analysis (Table 3) | 0 |

| | | | | |
|---|---|---|---|---|
| miR-187 | 1.23 | 0.0180 | Upregulated in the opium consumed human subjects with increase of TNF-α and IL-10 [153]. Upregulated in the mice- sepsis heart treated with mesenchymal stromal stem cells [50]. | 14 (e.g., *Rnaseh2a, Adcy1, Mplkip, Adcy1*) |
| miR-m107-1-5p | 1.23 | 0.0385 | Reported in the treatment of cytomegalovirus infection [154]. | 0 |
| miR-202-5p | 1.21 | 0.0121 | Induce macrophage apoptosis, release pro-inflammatory factors in the atherosclerotic plaque tissue of mice [155]. Modulating inflammation with TLR4 in SH-SY5Y cells [156]. | 8 (e.g., *Sox9, Onecut2, Fkbp9, Slc17a8, Irgq*) |
| miR-105 | 1.2 | 0.0100 | Modulator of TLR-2 protein translation in human gingival keratinocytes [157]. MI rat hearts injected with miR-105 had decreased infarct sizes [158]. | 81 (e.g., *Ceacam18, Agtrap, Coa5, Nhlrc2, Nfatc3*) |
| **miR-146b** | 1.19 | 0.0350 | Elevated with the progression of periodontal disease [159]. Potential biomarker in periodontal disease and diabetes [93]. | 23 (e.g., *Sirt1, Nfkb1, Zfp703, Slc47a1, Cd93*) |
| miR-m59-2 | 1.19 | 0.0434 | Augmented expression reported in splenocytes isolated from dicer deficient mice [160]. | 0 |
| miR-367 | 1.19 | 0.0436 | Reported as a therapeutic target in mice embryonal CNS-tumors [161]. Have a role in cardiomyocyte proliferation and development [162]. | 33 (e.g., Ncam2, Stk10, Fam136a, Cpeb1, Gm5148). |
| miR-201 | 1.18 | 0.0430 | Downregulated in the kidney of LPS-induced septic mice [163]. Cardioprotective effects by enhancing cardiomyocyte survival and function and attenuating cardiac fibrosis [164]. | 44 (e.g., *Thnsl1, St3gal2, Cd36, Whsc1l1, Mob3c*) |
| miR-33 | 1.17 | 0.0358 | Reported to regulate the lipid and glucose homeostasis [165]. Maintain the brown adipose tissue thermogenesis and whole-body metabolism for several stresses including cold stress [166]. Contributes to cardiometabolic diseases including obesity and atherosclerosis [167]. | 13 (e.g., *Ang, Crx, Dsg3, Celf2, Kcnj2*) |

miRNAs associated with periodontal disease are marked in red color.

**Table S6.** Comparison of downregulated miRNAs between 8-weeks and 16-weeks of infection.

| **miRNA** | **Fold change** | **p value** |
|---|---|---|
| miR-2135 | -2.19 | $1.8\times10^{-7}$ |
| miR-2133 | -2.13 | $4.7\times10^{-5}$ |
| miR-140 | -1.52 | $2.8\times10^{-5}$ |

| | | |
|---|---|---|
| miR-1937a+mmu-miR-1937b | -1.48 | 1.5×10$^{-3}$ |
| miR-1937c | -1.39 | 5.0×10$^{-4}$ |
| miR-720 | -1.36 | 2.6×10$^{-4}$ |
| miR-329 | -1.33 | 7.2×10$^{-3}$ |
| miR-338-3p | -1.31 | 7.5×10$^{-3}$ |
| miR-130a | -1.27 | 2.4×10$^{-3}$ |
| miR-322 | -1.27 | 1.0×10$^{-2}$ |
| miR-365 | -1.25 | 5.0×10$^{-3}$ |
| miR-2138 | -1.24 | 1.8×10$^{-2}$ |
| miR-362-3p | -1.22 | 4.0×10$^{-2}$ |
| miR-149 | -1.21 | 1.0×10$^{-2}$ |
| miR-99a | -1.19 | 1.7×10$^{-2}$ |
| miR-450a-5p | -1.19 | 1.8×10$^{-2}$ |
| miR-326 | -1.19 | 3.9×10$^{-2}$ |
| miR-342-3p | -1.18 | 2.6×10$^{-2}$ |
| miR-145 | -1.17 | 3.9×10$^{-3}$ |

**Table S7.** miRTarBase analysis of upregulated DE microRNAs and their target genes in 8 weeks of *S. gordonii* infection

| MiRTarBase ID | miRNA | Target gene |
|---|---|---|
| MIRT001666 | mmu-miR-375-3p | Insm1 |
| MIRT001667 | mmu-miR-375-3p | Mxi1 |
| MIRT001668 | mmu-miR-375-3p | Usp1 |
| MIRT001669 | mmu-miR-375-3p | C1qbp |
| MIRT001670 | mmu-miR-375-3p | Jak2 |
| MIRT001671 | mmu-miR-375-3p | Adipor2 |
| MIRT001672 | mmu-miR-375-3p | Ahr |
| MIRT002934 | mmu-miR-375-3p | Mtpn |
| MIRT004477 | mmu-miR-375-3p | Olfr75-ps1 |
| MIRT006091 | mmu-miR-375-3p | Yap1 |
| MIRT008821 | mmu-miR-375-3p | Elavl4 |
| MIRT576978 | mmu-miR-375-3p | Triml1 |
| MIRT583478 | mmu-miR-375-3p | Foxj3 |
| MIRT592835 | mmu-miR-375-3p | Akap2 |
| MIRT594832 | mmu-miR-375-3p | Pacsin1 |
| MIRT595136 | mmu-miR-375-3p | Lpin1 |
| MIRT595545 | mmu-miR-375-3p | Cnih4 |
| MIRT595637 | mmu-miR-375-3p | Atrn |
| MIRT595673 | mmu-miR-375-3p | Rpsa |
| MIRT743067 | mmu-miR-375 | Foxj3 |
| MIRT748787 | mmu-miR-375 | Lpin1 |

| | | |
|---|---|---|
| MIRT748906 | mmu-miR-375 | Cnih4 |
| MIRT749114 | mmu-miR-375 | Rpsa |
| MIRT749119 | mmu-miR-375 | Atrn |
| MIRT749538 | mmu-miR-375 | Pacsin1 |
| MIRT750088 | mmu-miR-375 | Akap2 |
| MIRT753581 | mmu-miR-375 | Triml1 |
| **MiRTarBase ID** | **miRNA** | **Target gene** |
| MIRT744386 | mmu-miR-142-5p | Itgb1bp3 |
| MIRT745202 | mmu-miR-142-5p | Tmem69 |
| MIRT745936 | mmu-miR-142-5p | Smtnl2 |
| MIRT747490 | mmu-miR-142-5p | Zfp874b |
| MIRT748056 | mmu-miR-142-5p | 4933432B09Rik |
| MIRT748276 | mmu-miR-142-5p | Six2 |
| MIRT748871 | mmu-miR-142-5p | Tapt1 |
| MIRT749006 | mmu-miR-142-5p | Abcg1 |
| MIRT749084 | mmu-miR-142-5p | Plcl1 |
| MIRT749094 | mmu-miR-142-5p | Srgap1 |
| MIRT749143 | mmu-miR-142-5p | Epha7 |
| MIRT749173 | mmu-miR-142-5p | Nr1d2 |
| MIRT749218 | mmu-miR-142-5p | Anapc11 |
| MIRT749243 | mmu-miR-142-5p | Ctage5 |
| MIRT749350 | mmu-miR-142-5p | Phf3 |
| MIRT749406 | mmu-miR-142-5p | Cwc25 |
| MIRT749678 | mmu-miR-142-5p | Cd28 |
| MIRT751120 | mmu-miR-142-5p | Sort1 |
| MIRT751459 | mmu-miR-142-5p | BC003965 |
| MIRT751995 | mmu-miR-142-5p | Slc35a1 |
| MIRT752233 | mmu-miR-142-5p | 5430427O19Rik |
| MIRT753002 | mmu-miR-142-5p | Sltm |
| MIRT753154 | mmu-miR-142-5p | Wnt11 |
| MIRT753471 | mmu-miR-142-5p | Pm20d2 |

| **miRNA** | **# of MiRTarBase IDs** | **# of Target genes** |
|---|---|---|
| miR-34b-5p | 354 | 354 |
| miR-142-5p | 24 | 24 |
| miR-135a | 19 | 19 |
| miR-323-3p | 25 | 25 |
| miR-485 | 14 | 14 |

We used mmu-miR-375 as an example for an upregulated DE miRNA during 8 weeks of infection in identifying the target genes using the miRTarBase. Each miRNA has different target genes and each with a specific MiRTarBase ID. *S. gordonii*-infection induced DE upregulated mmu-miR-375 has 27 different target genes with 27 different MiRTarBase ID as stated in the table. Similarly, the mmu-miR-142-5p has 24

different target genes with specific MiRTarBase ID. The other 5 of upregulated miRNAs and their # of target genes is stated in the separate table.

**Table S8.** miRTarBase analysis of upregulated DE microRNAs and their target genes in 16 weeks of *S. gordonii* infection**.**

| **MiRTarBase ID** | **miRNA** | **Target gene** |
|---|---|---|
| MIRT590085 | mmu-miR-1902 | Evi2b |
| MIRT591487 | mmu-miR-1902 | Arhgef15 |
| MIRT592674 | mmu-miR-1902 | Itga11 |
| MIRT594621 | mmu-miR-1902 | Myo9a |
| MIRT596881 | mmu-miR-1902 | Tsn |
| **miRNA** | **# of MiRTarBase IDs** | **# of Target genes** |
| miR-203 | 70 | 70 |
| miR-98 | 39 | 39 |
| miR-210 | 47 | 47 |
| miR-876-3p | 19 | 19 |
| mmu-let-7c | 37 | 37 |

The mmu-miR-1902 an upregulated DE miRNA during 16 weeks of *S. gordonii*-infection has 5 different target genes with 5 different MiRTarBase ID as stated in the table. The other 5 of upregulated miRNAs and their # of target genes is stated in the table.

**Table S9** Logistic Regression, C-Support Vector Machine, and Multilayer Perceptron most important agreed on miRNA features and reported miRNA functions.

| **miRNA** | **MIMAT #** | **Target functions** |
|---|---|---|
| | | **8 Weeks Analysis** |
| miR-22 | MIMAT0000531 | Promotes cell differentiation, tumor initiation, progression, and metastasis by maintaining Wnt/β-catenin signaling and cancer stem cells function[168]. Observed in the inflammatory mouse lung and brain tissues of polyinosinic-polycytidylic acid-treated mice. Upregulated in periodontal disease and obesity [99]. |
| miR-1 | MIMAT0000123 | Up-regulated in $H_2O_2$ treated cardiomyocytes as well as in miR-1 transgenic mice and MI mice [169] and in acute myocardial infarction [170] and altered in different cancer conditions [171]. Critical regulator of chondrocyte proliferation [172]; downregulated in the bones of Chinese osteoporotic patients [173]; downregulating the miR-1 promotes BMSCs osteogenic differentiation [174]. |
| miR-720 | MIMAT0003484 | Promotes glioma growth and upregulates invasion-related genes. Significantly upregulated in glioma tissues and cells [175]. |
| Let-7a | MIMAT0000521 | Proinflammatory role for let-7 miRNAs in experimental asthma [108]. |

| | | |
|---|---|---|
| | **16 Weeks Analysis** | |
| **miRNA** | **MIMAT #** | **Target functions** |
| miR-720 | MIMAT0003484 | Shown in the 8 weeks analysis of this table |
| let-7c | MIMAT0000523 | Interfere with critical inflammatory cytokine production viz., IL-1β, IL-6, and TNF-α in human Osteoarthritis (OA) and rheumatoid arthritis (RA) [102].Playing a role in cardiomyogenesis promotion activity [103]. |
| miR-1 | MIMAT0000123 | Shown in the 8 weeks analysis of this table |
| miR-205 | MIMAT0000238 | Refer 8 weeks analysis in the Table # 3 of main manuscript. |
| | **Combined 8 and 16 weeks** | |
| **miRNA** | **MIMAT #** | **Target functions** |
| miR-22 | MIMAT0000531 | Shown in the 8 weeks analysis of this table |
| miR-720 | MIMAT0003484 | Shown in the 8 weeks analysis of this table |
| let-7a | MIMAT0000521 | Shown in the 8 weeks analysis of this table |
| let-7c | MIMAT0000523 | Shown in the 16 weeks analysis of this table |
| miR-1 | MIMAT0000123 | Shown in the 8 weeks analysis of this table |

**Table S10.** List of miRNAs unique in expression among the DE and 5 ML models.

| miRNA | NanoString DE analysis | | | Machine Learning models | | | | |
|---|---|---|---|---|---|---|---|---|
| | 8 weeks | 16 weeks | 8 & 16 Weeks | XGB | RF | LR | SVC | MLP |
| miR-129-3p | ✓ | | ✓ | | | | | |
| miR-142-5p | ✓ | | | ✓ | ✓ | | | |
| miR-187 | ✓ | | ✓ | | | | | |
| miR-205 | ✓ | | ✓ | | | ✓ | ✓ | ✓ |
| miR-323-3p | ✓ | | | ✓ | ✓ | | | |
| miR-375 | ✓ | | ✓ | | | | | |
| miR-767 | ✓ | | ✓ | | | | | |
| let-7a | | | | | | ✓ | ✓ | ✓ |
| let-7c | | | | | | ✓ | ✓ | ✓ |
| miR-1 | | | | | | ✓ | ✓ | ✓ |
| miR-146b | | | ✓ | | | | | |
| miR-148a | ✓ | | | | | | ✓ | |
| miR-200c | ✓ | | ✓ | | | | | |
| miR-202-5p | ✓ | | ✓ | | | | | |

| | | | | | | | | |
|---|---|---|---|---|---|---|---|---|
| miR-203 | | ✓ | ✓ | | | | | |
| miR-210 | ✓ | ✓ | ✓ | | | | | |
| miR-22 | | | | | | ✓ | ✓ | ✓ |
| miR-23a | ✓ | | | | | ✓ | | |
| miR-30c | | | | ✓ | ✓ | | | |
| miR-33 | ✓ | | ✓ | | | | | |
| miR-339-5p | | | | ✓ | ✓ | | | |
| miR-345-3p | | | | ✓ | ✓ | | | |
| miR-367 | ✓ | | ✓ | | | | | |
| miR-423-5p | ✓ | ✓ | | | | | | |
| miR-449b | | | | ✓ | ✓ | | | |
| miR-455 | ✓ | | | | ✓ | | | |
| miR-590-5p | ✓ | | ✓ | | | | | |
| miR-720 | | | | | | ✓ | ✓ | ✓ |
| miR-m59-2 | ✓ | | | | ✓ | | | |

Red color (✓) indicates all three non-tree-based ML models identified miRs and blue color (✓) indicates two tree-based ML models identified miRs.

## APPENDICES

### Appendix S1: Hyperparameters for machine learning models

Information about the hyperparameters used for the machine models are available at:

- Logistic Regression (LR):
  https://scikit-learn.org/stable/modules/generated/sklearn.linear_model.LogisticRegression.html
- C-Support Vector Classifier:
  https://scikit-learn.org/stable/modules/generated/sklearn.svm.SVC.html#sklearn.svm.SVC
- Multilayer Perceptron (MLP):
  https://scikit-learn.org/stable/modules/generated/sklearn.neural_network.MLPClassifier.html
- Random Forest Classifier (RFC):
  https://scikit-learn.org/stable/modules/generated/sklearn.ensemble.RandomForestClassifier.html
- XGBoost (XGB):
  https://xgboost.readthedocs.io/en/stable/parameter.html

The following tables provide information about the hyperparameter settings used during the execution of each machine learning model on the data from 8-week, 16-week, and 8- and 16-week combined cohorts of mice.

**Logistic regression**

| Parameter | 8-week data | 16-week data | 8- and 16-week data |
|---|---|---|---|
| C | 0.1 | 10 | 1000 |
| max_iter | 50 | 50 | 200 |
| penalty | L2 | L2 | L1 |
| solver | newton-cg | newton-cg | liblinear |

**C-Support Vector Classifier**

| Parameter | 8-week data | 16-week data | 8- and 16-week data |
|---|---|---|---|
| C | 0.1 | 0.1 | 100 |
| gamma | 1 | 1 | scale |
| kernel | poly | poly | rbf |

**Multilayer Perceptron**

| Parameter | 8-week data | 16-week data | 8- and 16-week data |
|---|---|---|---|
| activation | relu | relu | relu |
| alpha | 0.01 | 0.01 | 0.01 |
| hidden_layer_sizes | (256, 128) | (400, 200) | (400,200) |
| learning_rate | adaptive | constant | constant |
| solver | adam | adam | adam |

**Random Forest Classifier**

| Parameter | 8-week data | 16-week data | 8- and 16-week data |
|---|---|---|---|
| bootstrap | False | False | True |
| criterion | gini | entropy | gini |
| max_depth | 90 | 20 | 20 |
| max_features | None | None | None |
| min_samples_leaf | 2 | 4 | 4 |
| min_samples_split | 8 | 2 | 8 |
| n_estimators | 290 | 20 | 60 |
| oob_score | False | False | False |
| random_state | 0 | 0 | 0 |

**XGBoost**

| Parameter | 8-week data | 16-week data | 8- and 16-week data |
|---|---|---|---|
| booster | gbtree | gbtree | gbtree |
| colsample_bytree | 0.6 | 0.4 | 0.6 |
| gamma | 0.2 | 0.3 | 0.2 |
| learning_rate | 0.5 | 0.1 | 0.5 |
| max_depth | 40 | 200 | 40 |
| n_estimators | 475 | 275 | 475 |
| objective | binary:logistic | binary:logistic | binary:logistic |
| random_state | 0 | 0 | 0 |
